# Early postoperative C-reactive protein-to-albumin ratio for risk stratification of osteomyelitis recurrence after surgical treatment

**DOI:** 10.3389/fmed.2026.1953342

**Published:** 2026-09-07

**Authors:** Muhammed Kazez, Anil Agar, Sefa Key, Hamza Yavuz, Ilyas Turk

**Affiliations:** 1 Department of Orthopedics and Traumatology, Elazig Fethi Sekin City Hospital, Elazig, Türkiye; 2 Department of Orthopedics and Traumatology, Faculty of Medicine, Firat University, Elazig, Türkiye

**Keywords:** C-reactive protein-to-albumin ratio, osteomyelitis, prognostic biomarker, recurrence, risk stratification, surgical treatment

## Abstract

**Objective:**

Reliable biomarkers for identifying patients at increased risk of osteomyelitis recurrence during the early postoperative period are lacking. The C-reactive protein-to-albumin ratio (CAR), which integrates systemic inflammatory burden and nutritional status, has shown diagnostic value in musculoskeletal infections, but its prognostic role after surgical treatment of osteomyelitis remains unclear. This study evaluated whether early postoperative CAR predicts osteomyelitis recurrence and compared its performance with conventional inflammatory biomarkers.

**Patients and methods:**

This retrospective cohort study included 90 consecutive adults who underwent surgical treatment for osteomyelitis between January 2020 and December 2024. Serum inflammatory biomarkers were recorded before surgery and at the first postoperative outpatient visit, approximately one month after surgery. The primary outcome was osteomyelitis recurrence during a minimum follow-up of 12 months. Receiver operating characteristic (ROC) analysis was used to evaluate the predictive performance of inflammatory biomarkers, and multivariable logistic regression was used to evaluate factors independently associated with recurrence.

**Results:**

During a mean follow-up of 19 months, recurrence occurred in 19 patients (21.1%). Patients with recurrence had significantly higher baseline and one-month CAR values than those without recurrence (both *p* < 0.001). Among the evaluated biomarkers, one-month CAR demonstrated the numerically highest AUC for recurrence (AUC = 0.862; optimal cutoff = 1.41), with a sensitivity of 73.7% and specificity of 87.3%. However, its AUC was not significantly different from that of one-month CRP (AUC = 0.831; DeLong *p* = 0.223). In multivariable analysis, one-month CAR remained independently associated with recurrence within the parsimonious multivariable model (OR 5.42, 95% CI 2.38–12.36; *p* < 0.001), together with the Charlson Comorbidity Index (OR 1.94, 95% CI 1.27–2.96; *p* = 0.002). Leukocyte-derived inflammatory indices showed substantially lower prognostic performance during postoperative follow-up.

**Conclusion:**

Early postoperative CAR was associated with osteomyelitis recurrence and showed good discriminative ability, with the numerically highest AUC among the evaluated inflammatory biomarkers. However, its discriminative performance was not statistically superior to that of one-month CRP. CAR may therefore represent a practical adjunctive biomarker for postoperative risk stratification. Prospective multicenter studies are warranted to validate these findings and determine whether CAR-guided surveillance improves patient outcomes.

## Introduction

Osteomyelitis remains one of the most challenging musculoskeletal infections despite substantial advances in surgical techniques, antimicrobial therapy, and multidisciplinary patient management. The disease is characterized by progressive bone destruction, prolonged treatment, repeated surgical interventions, functional impairment, and considerable healthcare costs. Although modern treatment strategies have significantly improved infection control, recurrence continues to represent a major obstacle to successful management, with reported recurrence rates ranging from 10% to over 30% depending on patient characteristics, anatomical location, microbiological profile, and treatment strategy (1–4). Identifying patients at increased risk of recurrence remains clinically important because delayed recognition of persistent infection may result in repeated operations, prolonged antibiotic therapy, chronic disability, or even limb amputation (5).

The diagnosis and follow-up of osteomyelitis rely on a combination of clinical assessment, laboratory investigations, microbiological findings, and imaging modalities. Bone biopsy with microbiological culture remains the diagnostic gold standard; however, its invasive nature limits its role during routine postoperative follow-up (6). Likewise, magnetic resonance imaging and nuclear imaging techniques provide valuable diagnostic information but may be difficult to interpret after surgery because postoperative inflammatory changes often persist for several months (7). Consequently, serum inflammatory biomarkers continue to play an important role in daily clinical practice because they are inexpensive, rapidly available, and routinely measured. Among these, C-reactive protein (CRP) and erythrocyte sedimentation rate (ESR) are the most widely used inflammatory markers. Nevertheless, both biomarkers have well-recognized limitations, including relatively low specificity and susceptibility to numerous inflammatory and systemic conditions unrelated to bone infection (8, 9).

Growing recognition of these limitations has led investigators to evaluate composite inflammatory biomarkers that combine different aspects of the host response into a single prognostic indicator. In recent years, several hematological indices—including the neutrophil-to-lymphocyte ratio (NLR), platelet-to-lymphocyte ratio (PLR), lymphocyte-to-monocyte ratio (LMR), systemic immune-inflammation index (SII), and C-reactive protein-to-albumin ratio (CAR)—have been investigated as potential biomarkers in infectious, inflammatory, cardiovascular, and oncological diseases (10–13). Compared with conventional inflammatory markers, these composite indices may better reflect the interaction between systemic inflammation, immune response, and nutritional status.

Among these biomarkers, CAR has attracted increasing attention because it integrates two routinely available laboratory parameters with complementary biological significance. CRP is a positive acute-phase reactant synthesized by hepatocytes in response to inflammatory cytokines, whereas albumin is a negative acute-phase protein whose concentration decreases during systemic inflammation and poor nutritional status. Consequently, CAR simultaneously reflects inflammatory burden and physiological reserve, potentially providing a more comprehensive assessment of disease activity than either parameter alone (14, 15). Elevated CAR has consistently been associated with adverse outcomes in patients with sepsis, bloodstream infections, coronavirus disease, malignancies, cardiovascular disorders, and critically ill patients (16–19).

Interest in CAR has recently expanded to orthopedic surgery. Previous studies have demonstrated promising diagnostic or prognostic value of CAR in prosthetic joint infection, diabetic foot osteomyelitis, fracture-related infection, periprosthetic fracture, and other orthopedic conditions (3, 20–24). However, most published studies have focused primarily on diagnostic performance at presentation rather than prediction of long-term clinical outcomes following treatment. Moreover, available studies evaluating musculoskeletal infections are limited by heterogeneous patient populations, different definitions of treatment success, and inconsistent timing of laboratory assessment. Consequently, the role of CAR during the postoperative period, particularly as an early predictor of osteomyelitis recurrence, remains insufficiently investigated.

From a biological perspective, evaluating CAR after surgery may provide clinically meaningful information. Successful surgical debridement combined with appropriate antimicrobial therapy should result in rapid resolution of systemic inflammation and gradual normalization of serum albumin concentrations. Conversely, persistent elevation of CRP accompanied by delayed albumin recovery may reflect ongoing inflammatory activity despite apparent clinical improvement. Because both laboratory parameters are routinely obtained during postoperative follow-up, CAR represents an inexpensive, reproducible, and easily applicable biomarker that requires no additional laboratory testing or specialized equipment.

Another important consideration is that recurrence of osteomyelitis differs fundamentally from persistent infection. While persistent infection generally reflects incomplete eradication of microorganisms during the initial treatment period, recurrence develops after an apparent period of clinical recovery and may result from residual bacterial biofilm, compromised vascularity, host-related factors, or inadequate local immune control (25, 26). Identifying patients who remain at increased risk during the early postoperative period remains challenging because conventional inflammatory markers frequently normalize despite persistent low-grade infection. Therefore, biomarkers capable of detecting subtle ongoing inflammatory activity may improve postoperative surveillance and facilitate individualized follow-up strategies.

Rather than relying solely on laboratory parameters obtained at the time of diagnosis, early post-treatment inflammatory biomarkers may better reflect the combined effects of surgical source control, antimicrobial therapy, and host immune recovery. Despite growing interest in inflammatory ratios across multiple medical specialties, evidence regarding the prognostic significance of early postoperative CAR in osteomyelitis remains scarce. Furthermore, few studies have directly compared postoperative CAR with conventional inflammatory markers in predicting recurrence after surgical treatment.

Therefore, the present study aimed to investigate the association between the early post-treatment C-reactive protein-to-albumin ratio and osteomyelitis recurrence in surgically treated patients. In addition, we compared the predictive performance of CAR with conventional inflammatory biomarkers and evaluated whether postoperative CAR could contribute to risk stratification during routine clinical follow-up. We hypothesized that an elevated early post-treatment CAR would be associated with an increased risk of osteomyelitis recurrence and might serve as a simple, readily available adjunctive biomarker for postoperative surveillance.

## Patients and methods

### Study design and patient selection

This retrospective cohort study was conducted at the Department of Orthopaedic Surgery of a tertiary referral university hospital. Consecutive adult patients who underwent surgical treatment for osteomyelitis between January 2020 and December 2024 were retrospectively identified from the institutional electronic medical record system. The study protocol was approved by the Institutional Non-Interventional Clinical Research Ethics Committee (approval number: 34463/14.05.2025), and the study was conducted in accordance with the ethical principles of the Declaration of Helsinki. Owing to the retrospective design, the requirement for informed consent was waived.

The study was designed and reported according to the Strengthening the Reporting of Observational Studies in Epidemiology (STROBE) recommendations for observational cohort studies. Patients were enrolled consecutively to minimize selection bias.

Adult patients (≥18 years) who underwent surgical treatment for osteomyelitis during the study period were eligible for inclusion.

The diagnosis of osteomyelitis was established using a combination of clinical findings, laboratory investigations, radiological imaging, intraoperative observations, microbiological culture results, and histopathological examination when available. Because the primary aim of the study was to evaluate the prognostic value of postoperative inflammatory biomarkers in routine orthopedic practice, patients with different etiologies of osteomyelitis were included. Accordingly, post-traumatic, postoperative, implant-associated, diabetic foot-related, hematogenous, contiguous, and vertebral osteomyelitis cases were eligible for inclusion, provided that surgical treatment had been performed and complete clinical, laboratory, and follow-up data were available.

To ensure a reliable assessment of recurrence, only patients who completed a minimum follow-up of 12 months after the initial treatment were included in the study. Patients were excluded if they had a follow-up duration of less than 12 months, incomplete demographic, clinical, or laboratory data, active malignancy, autoimmune or systemic inflammatory disease, chronic liver disease likely to affect serum albumin concentrations, or ongoing immunosuppressive therapy.

### Clinical management and data collection

Treatment decisions were made by the attending orthopedic surgeons according to the clinical presentation and extent of infection. Surgical intervention was indicated in patients with necrotic bone, chronic draining sinus, abscess formation, implant-related infection, progressive bone destruction, failure of previous conservative treatment, or persistent infection requiring operative debridement. All surgical procedures were performed by the same orthopedic surgical team according to standardized institutional treatment principles.

All patients underwent radical surgical debridement with excision of necrotic bone and devitalized soft tissue until viable bleeding tissue was encountered. Whenever feasible, multiple deep tissue specimens were obtained intraoperatively before antibiotic administration and submitted for microbiological analysis. Additional procedures, including implant removal, fracture stabilization, dead-space management, bone reconstruction, or soft tissue coverage, were performed according to the characteristics of each individual case and the operating surgeon's judgement. Empirical intravenous antibiotic therapy was initiated after intraoperative sampling and subsequently modified according to microbiological culture results and antimicrobial susceptibility testing in consultation with infectious disease specialists. The duration of antimicrobial therapy was individualized according to the causative microorganism, clinical response, and institutional treatment protocols.

Demographic characteristics, clinical variables, operative records, microbiological findings, laboratory results, and follow-up data were retrieved from the institutional electronic database and individual patient records. Recorded demographic variables included age, sex, smoking status, and Charlson Comorbidity Index (CCI). Clinical variables included the etiology of osteomyelitis, anatomical location, and isolated microorganisms. Osteomyelitis etiology was categorized as post-traumatic, implant-associated following fracture fixation, postoperative, diabetic foot-related, hematogenous, or contiguous spread from adjacent soft tissue infection, while anatomical localization was recorded according to the involved bone.

### Laboratory assessment

Routine laboratory investigations were performed at two predefined time points: immediately before surgical treatment and during the first scheduled postoperative outpatient visit, approximately one month after surgery. The median interval between surgery and postoperative blood sampling was 31 days (IQR, 28–34; range, 25–37 days). For the purposes of the study, laboratory measurements obtained within 25–37 days after surgery were considered the one-month postoperative assessment. Serum C-reactive protein (CRP), albumin, erythrocyte sedimentation rate (ESR), white blood cell count, neutrophil count, lymphocyte count, monocyte count, and platelet count were recorded at both evaluations.

The C-reactive protein-to-albumin ratio (CAR) was calculated by dividing serum CRP concentration (mg/L) by serum albumin concentration (g/dL). This unit convention was applied consistently for all CAR calculations and subsequent statistical analyses. In addition, neutrophil-to-lymphocyte ratio (NLR), platelet-to-lymphocyte ratio (PLR), monocyte-to-lymphocyte ratio (MLR), derived neutrophil-to-lymphocyte ratio (dNLR), systemic inflammatory response index (SIRI), and systemic immune-inflammation index (SII) were calculated using standard formulas. Both baseline and early postoperative values were analyzed to determine whether dynamic changes in inflammatory biomarkers were associated with subsequent osteomyelitis recurrence.

### Follow-up and outcome measures

Patients were followed according to the routine postoperative protocol used at our institution, as described in the original study. Clinical examination, laboratory investigations, and standard radiographic evaluation were performed at each follow-up visit, whereas advanced imaging studies were obtained when recurrent infection was clinically suspected.

The primary outcome of the study was osteomyelitis recurrence. Recurrence was defined as the reappearance of clinical signs or symptoms consistent with infection following completion of the initial treatment, together with at least one objective finding, including positive microbiological culture, radiological evidence of recurrent osteomyelitis, or the requirement for repeat surgical debridement because of confirmed recurrent infection. Patients who remained free of clinical, laboratory, and radiological evidence of infection throughout follow-up were classified as the non-recurrence group.

### Statistical analysis

Statistical analyses were performed using IBM SPSS Statistics for Windows, version 25.0 (IBM Corp., Armonk, NY, USA). The distribution of continuous variables was assessed using the Shapiro–Wilk test and by visual inspection of histograms and probability plots. Normally distributed variables were presented as mean ± standard deviation, whereas variables with a non-normal distribution were reported as median and interquartile range. Categorical variables were summarized as frequencies and percentages.

Patients were classified into recurrence and non-recurrence groups according to the predefined recurrence criteria described above. Continuous variables were compared between the two groups using the independent-samples Student's t-test when the assumptions of normality were met and the Mann–Whitney U test when these assumptions were not met. Categorical variables were compared using the Pearson chi-square test or Fisher's exact test, depending on the expected cell frequencies. In exploratory analyses, CAR values were also compared according to implant-associated etiology and anatomical location. The Mann–Whitney U test was used to compare implant-associated and non-implant-associated cases, whereas the Kruskal–Wallis test was used for comparisons across anatomical locations.

Univariable logistic regression analyses were performed to examine the association between the recorded demographic, clinical, microbiological, and laboratory variables and osteomyelitis recurrence. Because only 19 recurrence events occurred, the multivariable analysis was intentionally restricted to a parsimonious model to reduce the risk of overfitting. One-month CAR, which was the principal biomarker of interest and showed the numerically highest AUC, and the Charlson Comorbidity Index, which represented baseline host comorbidity burden, were included in the final multivariable binary logistic regression model. A CAR   ×   CCI interaction term was additionally evaluated in an exploratory analysis to assess whether the association between postoperative CAR and recurrence differed according to baseline comorbidity burden. One-month CAR was entered into the logistic regression model as a continuous variable rather than dichotomized according to the ROC-derived cutoff. The assumption of linearity in the logit for one-month CAR was assessed using the Box–Tidwell approach by introducing an interaction term between CAR and its natural logarithm. CRP and albumin were not entered into the same model as CAR because CAR is directly derived from these variables and their simultaneous inclusion could introduce multicollinearity. Similarly, NLR and MLR, despite showing significant associations with recurrence in univariable analyses, were not included in the final multivariable model because the limited number of recurrence events (*n* = 19) necessitated restriction of the number of predictors to minimize model instability and overfitting. Baseline and one-month CAR values were also not entered together in the primary model because of their biological and statistical correlation. Regression results were reported as odds ratios with corresponding 95% confidence intervals.

Receiver operating characteristic curve analysis was used to assess the ability of inflammatory biomarkers and the Charlson Comorbidity Index to discriminate between patients with and without recurrence. The area under the receiver operating characteristic curve and its 95% confidence interval were calculated for each variable. Optimal cutoff values were identified using the Youden index, and the corresponding sensitivity, specificity, positive predictive value, and negative predictive value were calculated. The discriminative performance of one-month CAR and one-month CRP was additionally compared using the DeLong test for correlated ROC curves. Internal validation of the discriminatory performance of one-month CAR was performed using 2,000 bootstrap resamples. Bootstrap resampling was used to assess the stability of the AUC and the ROC-derived optimal cutoff. All statistical tests were two-sided, and a *p* value of less than 0.05 was considered statistically significant.

## Results

### Patient characteristics

**Figure 1:**
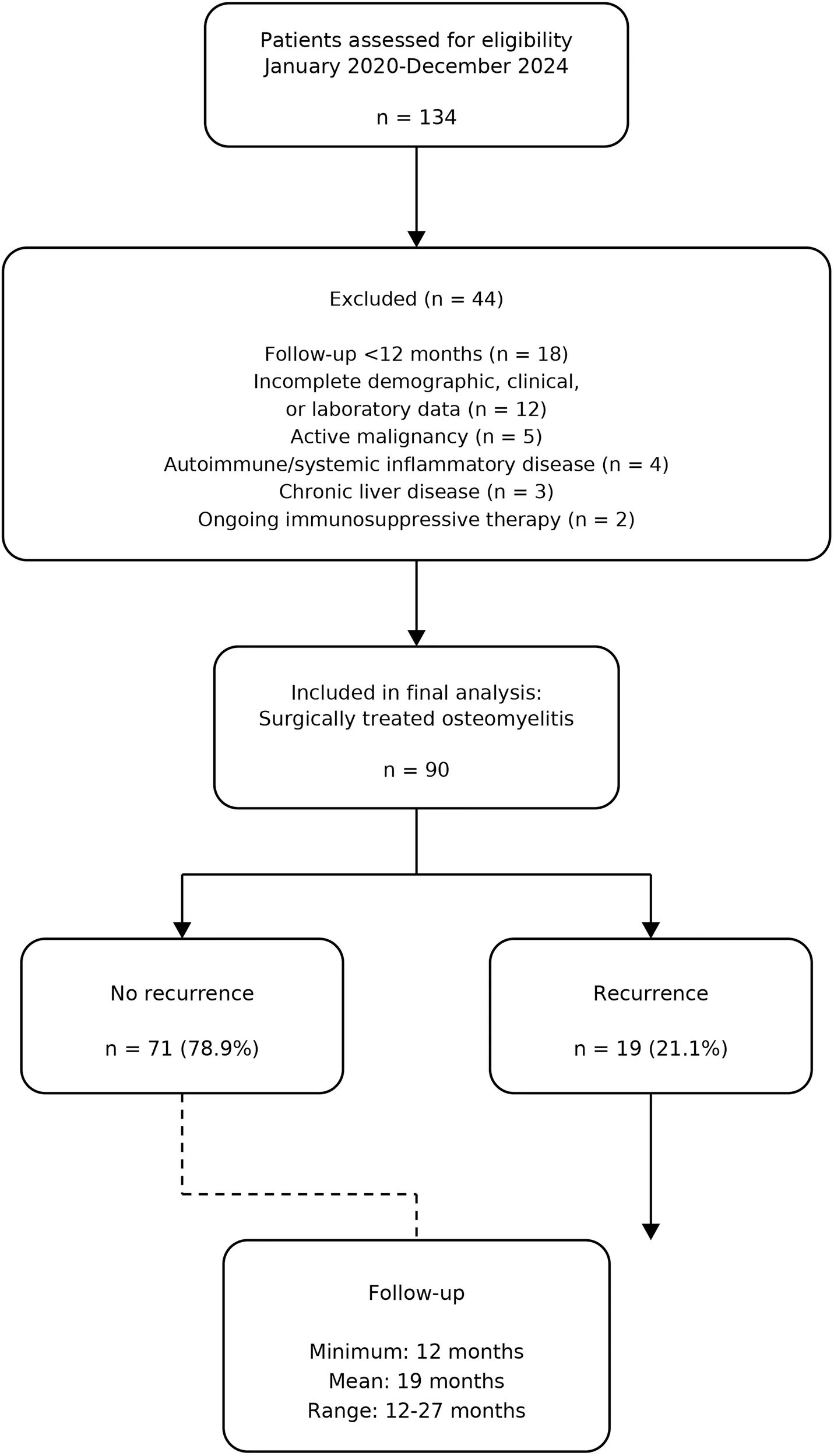
Flow diagram of patient selection. Flowchart illustrating patient screening, application of inclusion and exclusion criteria, and the final study cohort.

A total of 134 patients were screened for eligibility during the study period. After applying the predefined inclusion and exclusion criteria, 90 patients were included in the final analysis (Figure 1). The mean follow-up duration was 19 months (range, 12–27 months). During follow-up, osteomyelitis recurrence occurred in 19 patients (21.1%), whereas 71 patients (78.9%) remained recurrence-free. The median interval between surgery and postoperative laboratory assessment was 31 days (IQR, 28–34; range, 25–37 days).

The study population represented a heterogeneous spectrum of surgically treated osteomyelitis, including diabetic foot, implant-related, vascular insufficiency-associated, and post-traumatic infections involving different anatomical locations. Culture positivity was observed in 61 patients (67.8%), with *methicillin-sensitive Staphylococcus aureus* being the most frequently isolated microorganism.

Baseline demographic characteristics, osteomyelitis etiology, anatomical distribution, and microbiological findings were generally comparable between the recurrence and non-recurrence groups. No significant between-group differences were observed regarding age, sex, smoking status, osteomyelitis etiology, anatomical location, culture positivity, or isolated microorganisms (all *p* > 0.05). Detailed baseline characteristics are presented in Table 1.

**Table 1:** Baseline demographic, clinical, microbiological and osteomyelitis characteristics according to recurrence status.

Variable	Overall (*n* = 90)	No recurrence (*n* = 71)	Recurrence (*n* = 19)	*p*-value
Age, years	38 (22–55)	33 (22–59)	39 (26–55)	0.667
Male sex, *n* (%)	55 (61.1)	45 (63.4)	10 (52.6)	0.434
Smoking, *n* (%)	52 (57.8)	43 (60.6)	9 (47.4)	0.311
Charlson Comorbidity Index	1.0 (0.0–3.0)	1.0 (0.0–2.0)	3.0 (2.0–4.0)	<0.001
Diabetic foot osteomyelitis	34 (37.8)	25 (35.2)	9 (47.4)	0.768
Implant-related osteomyelitis	30 (33.3)	25 (35.2)	5 (26.3)	
Vascular insufficiency-associated osteomyelitis	19 (21.1)	15 (21.1)	4 (21.1)	
Post-traumatic osteomyelitis	7 (7.8)	6 (8.5)	1 (5.3)	
Foot	36 (40.0)	29 (40.8)	7 (36.8)	0.406
Tibia	24 (26.7)	16 (22.5)	8 (42.1)	
Femur	11 (12.2)	9 (12.7)	2 (10.5)	
Upper extremity	14 (15.6)	12 (16.9)	2 (10.5)	
Vertebral column	5 (5.6)	5 (7.0)	0 (0.0)	
Culture-positive infection, *n* (%)	61 (67.8)	51 (71.8)	10 (52.6)	0.166
MSSA	20 (22.2)	17 (23.9)	3 (15.8)	0.179
MRSA	4 (4.4)	2 (2.8)	2 (10.5)	
*Staphylococcus epidermidis*	15 (16.7)	14 (19.7)	1 (5.3)	
Gram-negative bacilli	12 (13.3)	11 (15.5)	1 (5.3)	
Polymicrobial	10 (11.1)	7 (9.9)	3 (15.8)	
Culture negative	29 (32.2)	20 (28.2)	9 (47.4)	

Values are presented as median (interquartile range) or *n* (%), as appropriate. *P*-values were calculated using Mann–Whitney U test, chi-square test, or Fisher's exact test. The reported *p*-value represents the overall comparison among osteomyelitis etiologies.

### Comparison of laboratory parameters

**Figure 2:**
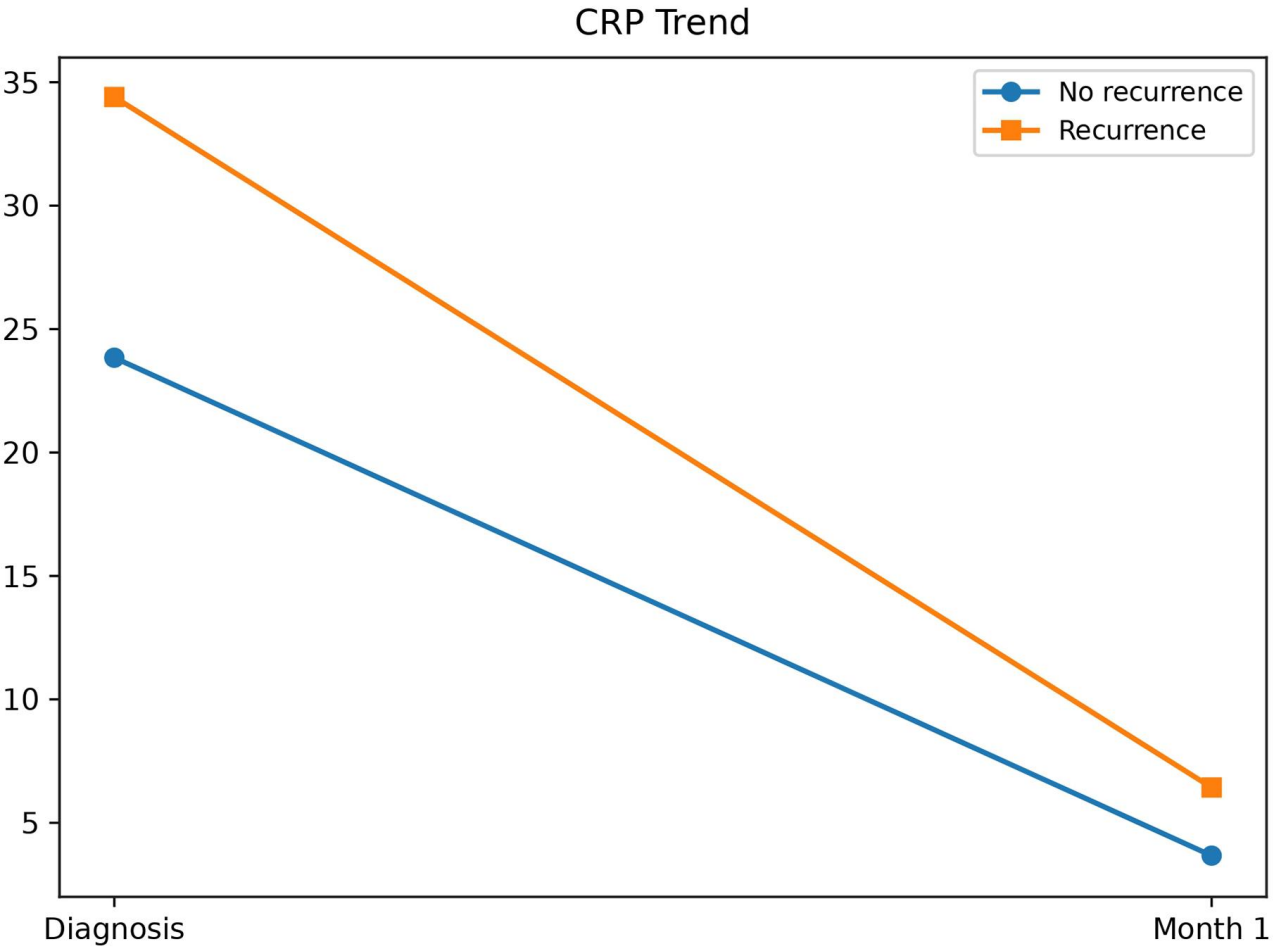
Temporal changes in CRP from diagnosis to one month after surgery according to recurrence status.

Patients who developed recurrence demonstrated persistently greater systemic inflammatory activity than those who remained recurrence-free. At the time of diagnosis, recurrence was associated with significantly higher CRP levels and lower serum albumin concentrations (both *p* < 0.001). Similar differences persisted at the one-month follow-up evaluation, with the recurrence group continuing to exhibit significantly higher CRP levels and lower albumin concentrations than the non-recurrence group (both *p* < 0.001) (Figure 2).

Among leukocyte-derived inflammatory indices, baseline NLR and MLR were significantly higher in patients who subsequently developed recurrence (*p* = 0.009 and *p* = 0.040, respectively). However, no significant differences were observed between the groups for one-month NLR, PLR, MLR, or dNLR values.

Patients with recurrence also had a significantly higher Charlson Comorbidity Index than those without recurrence (median, 3 vs. 1; *p* < 0.001).

**Figure 3:**
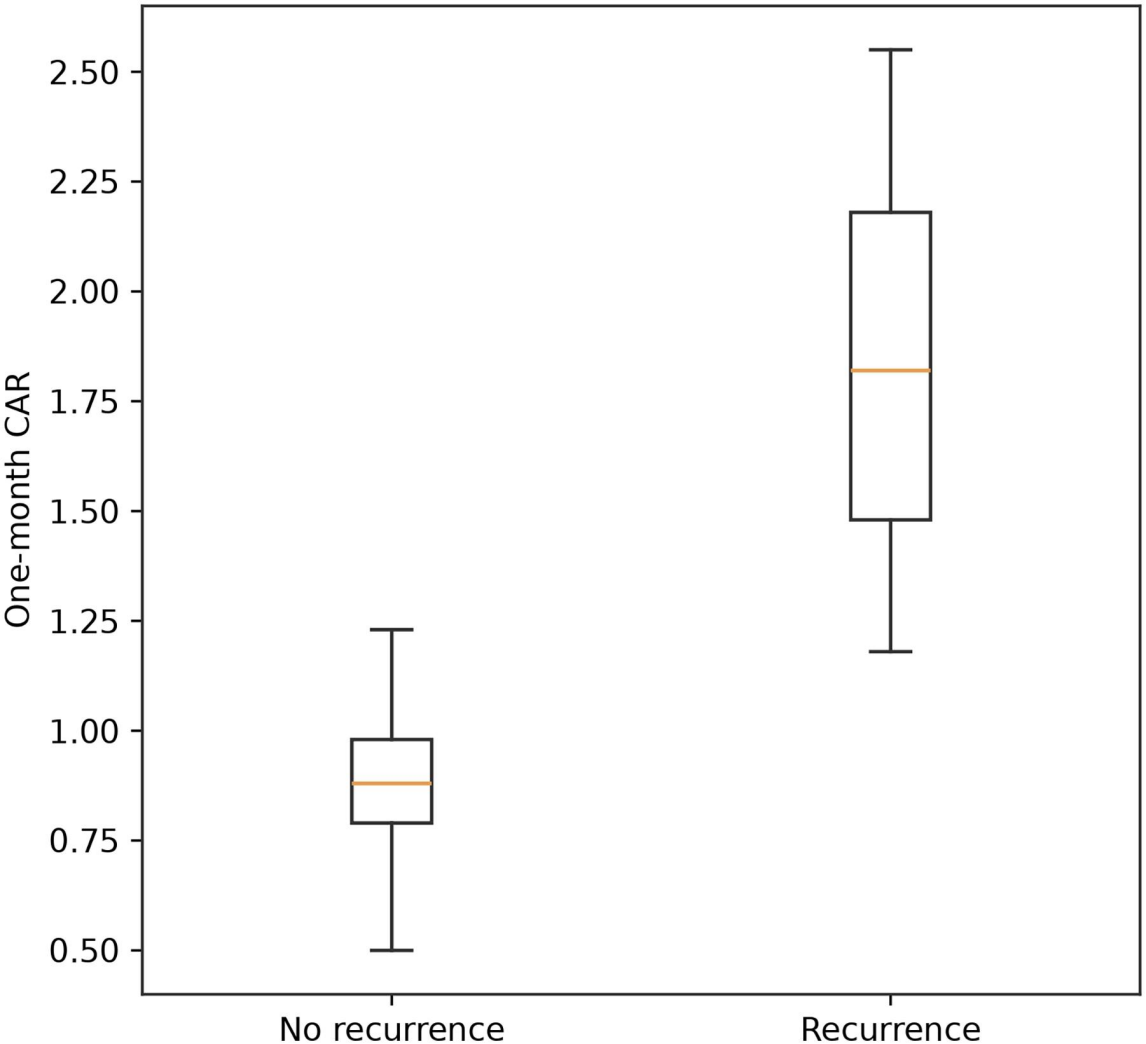
Distribution of one-month CRP-to-albumin ratio (CAR) according to recurrence status. Patients with recurrence demonstrated significantly higher CAR values than those without recurrence.

**Figure 4:**
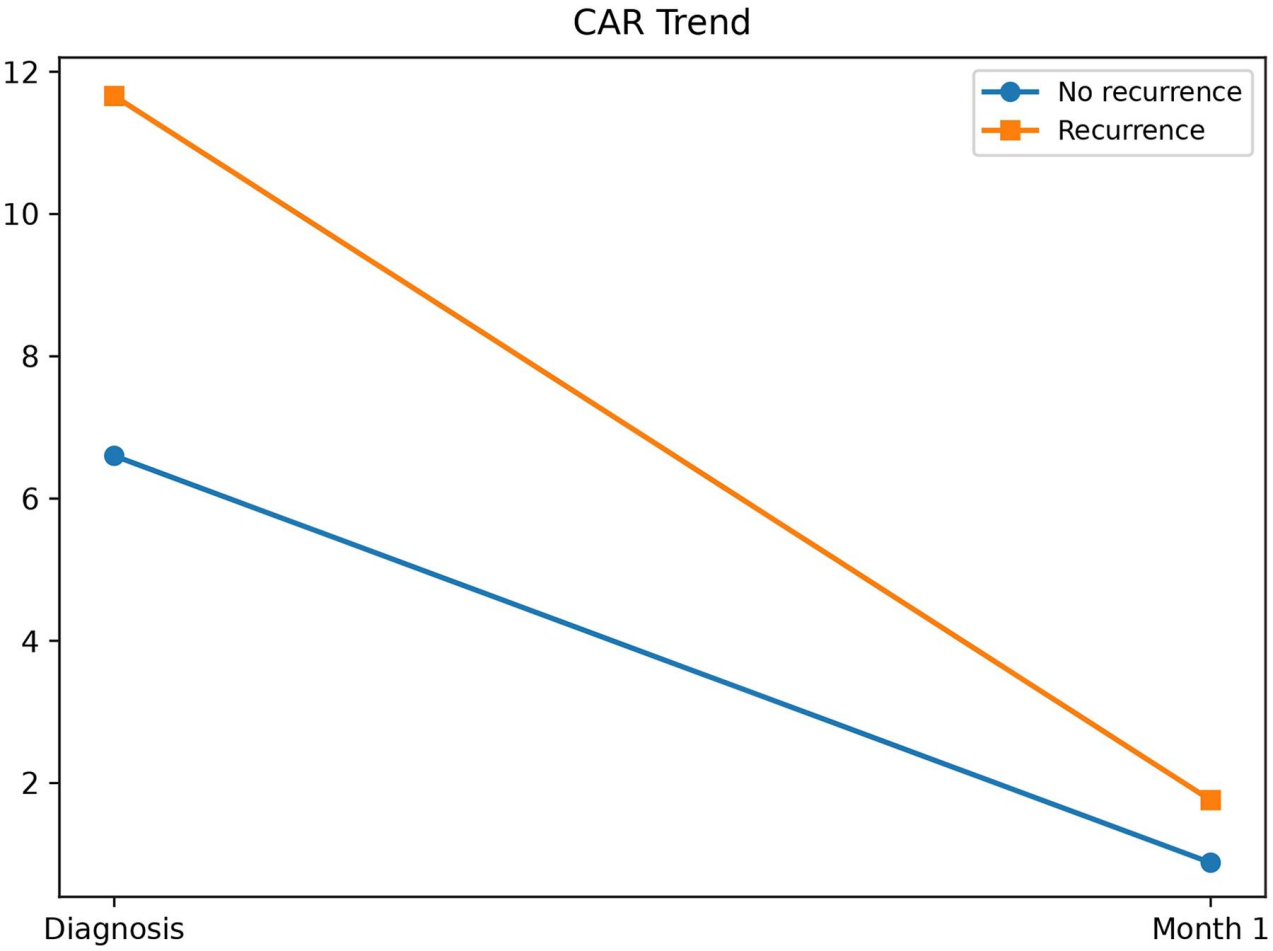
Changes in CRP-to-albumin ratio (CAR) from diagnosis to one month according to recurrence status. Persistent elevation of CAR was observed in patients who subsequently developed recurrence.

The C-reactive protein-to-albumin ratio showed the most consistent association with recurrence. Both baseline and one-month CAR values were significantly higher in the recurrence group, with the greatest between-group difference observed at the one-month assessment (both *p* < 0.001) (Figures 3, 4). Complete comparisons of laboratory parameters and inflammatory biomarkers are presented in Tables 2, 3.

**Table 2:** Baseline inflammatory biomarkers according to recurrence status.

Variable	Overall (*n* = 90)	No recurrence (*n* = 71)	Recurrence (*n* = 19)	*p*-value
CRP, mg/L	26.16 (20.02–33.37)	23.84 (17.54–30.18)	34.40 (30.52–43.86)	**<0**.**001**
Albumin, g/dL	3.37 (3.06–3.72)	3.45 (3.13–3.83)	3.06 (2.85–3.36)	**<0**.**001**
CAR	7.03 (5.26–9.59)	6.60 (4.91–9.02)	11.66 (9.17–15.08)	**<0**.**001**
NLR	4.48 (2.93–7.65)	3.72 (2.83–6.99)	7.28 (4.48–8.88)	0.009
PLR	163.84 (134.18–218.58)	162.31 (122.04–218.49)	193.37 (136.83–223.07)	0.566
MLR	0.43 (0.30–0.63)	0.40 (0.28–0.62)	0.59 (0.40–0.76)	0.040
dNLR	2.47 (1.84–3.15)	2.31 (1.79–2.99)	2.83 (2.28–3.20)	0.078

Values are presented as median (interquartile range).

CAR, C-reactive protein-to-albumin ratio; NLR, neutrophil-to-lymphocyte ratio; PLR, platelet-to-lymphocyte ratio; MLR, monocyte-to-lymphocyte ratio; dNLR, derived neutrophil-to-lymphocyte ratio.

**Table 3:** One-month inflammatory biomarkers according to recurrence status.

Variable	Overall (*n* = 90)	No recurrence (*n* = 71)	Recurrence (*n* = 19)	*p*-value
CRP, mg/L	4.00 (3.14–5.30)	3.67 (3.00–4.51)	6.43 (4.59–10.62)	**<0**.**001**
Albumin, g/dL	3.98 (3.51–4.16)	4.06 (3.83–4.20)	3.27 (3.18–3.62)	**<0**.**001**
CAR	1.01 (0.75–1.38)	0.88 (0.69–1.21)	1.76 (1.31–3.32)	**<0**.**001**
NLR	3.69 (2.21–5.41)	3.72 (2.19–5.59)	3.65 (2.24–4.81)	0.953
PLR	167.30 (133.86–223.68)	165.91 (124.99–220.81)	195.88 (141.24–231.55)	0.353
MLR	0.21 (0.18–0.24)	0.21 (0.19–0.23)	0.23 (0.18–0.24)	0.355
dNLR	1.89 (1.46–2.34)	1.99 (1.38–2.34)	1.69 (1.59–2.33)	0.851

Values are presented as median (interquartile range). One-month values were obtained at the postoperative assessment performed 25–37 days after surgery.

Bold values indicate statistical significance (*p* < 0.05).

In exploratory analyses, CAR values did not differ significantly between implant-associated and non-implant-associated osteomyelitis. Median one-month CAR was 1.03 (IQR, 0.81–1.47) in implant-associated cases and 1.00 (IQR, 0.75–1.29) in non-implant-associated cases (*p* = 0.761). Similarly, no significant differences in one-month CAR were observed across anatomical locations (*p* = 0.209). Baseline CAR also did not differ significantly according to implant-associated etiology (*p* = 0.817) or anatomical location (*p* = 0.847).

### Receiver operating characteristic analysis

**Figure 5:**
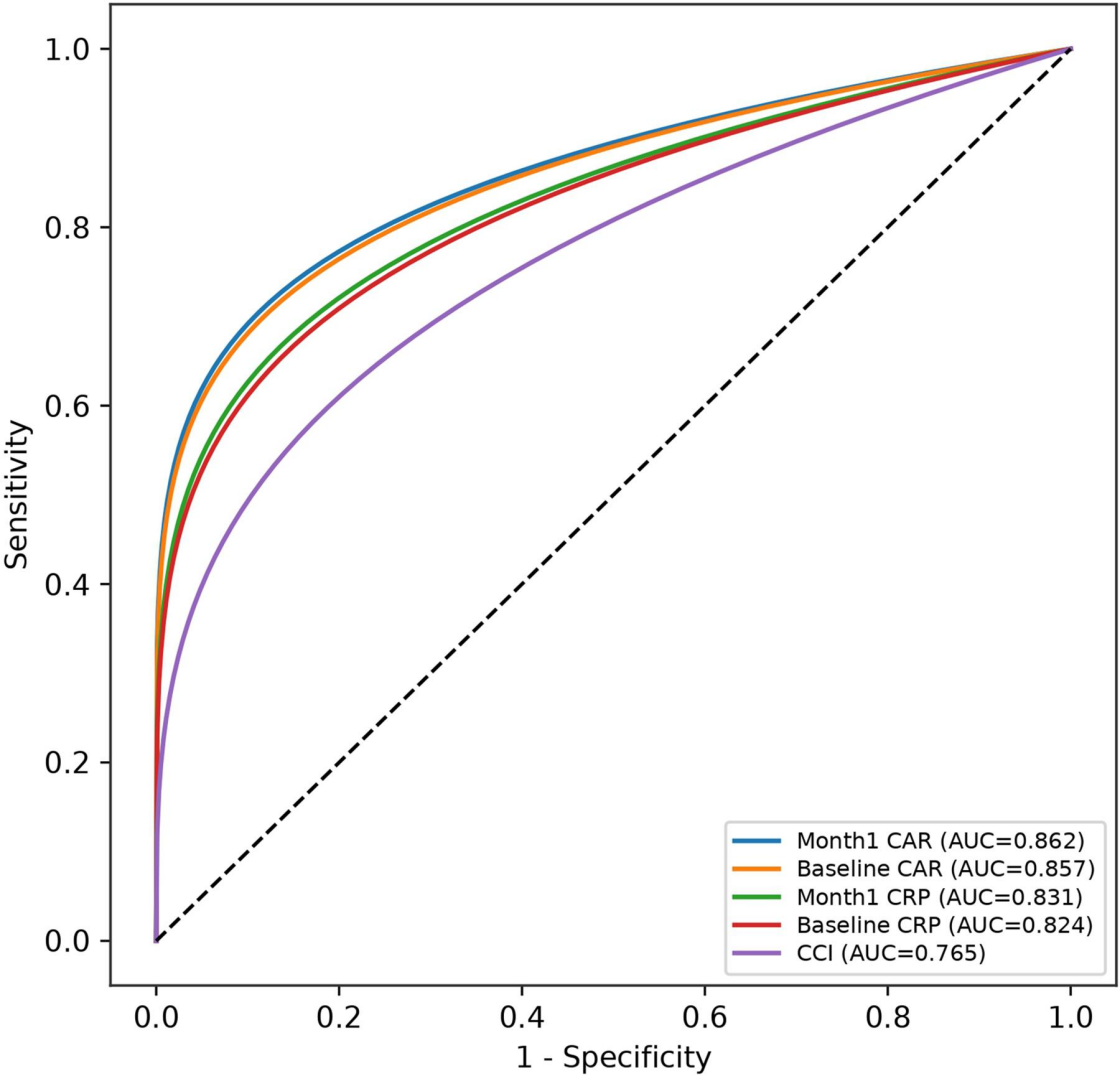
Receiver operating characteristic (ROC) curves for predicting osteomyelitis recurrence. One-month CAR demonstrated the numerically highest AUC among the evaluated biomarkers.

Receiver operating characteristic (ROC) analysis demonstrated that one-month CAR had the numerically highest AUC among the evaluated biomarkers (Figure 5). The AUC for one-month CAR was 0.862, compared with 0.831 for one-month CRP, 0.857 for baseline CAR, 0.824 for baseline CRP, 0.840 for albumin, and 0.765 for CCI. However, pairwise comparison of the ROC curves using the DeLong test showed that the difference in AUC between one-month CAR and one-month CRP was not statistically significant (*Δ*AUC = 0.030; *p* = 0.223). Internal validation using 2,000 bootstrap resamples demonstrated a median AUC of 0.863 (95% bootstrap percentile interval, 0.763–0.943). The median bootstrap-derived optimal cutoff was 1.407, although the corresponding 95% percentile interval was relatively wide (0.870–1.762), indicating greater variability in the estimated optimal threshold than in overall discriminatory performance.

The optimal cutoff value for one-month CAR, determined using the Youden index, was 1.41, corresponding to a sensitivity of 73.7% and a specificity of 87.3%. This cutoff should be regarded as an exploratory, cohort-derived threshold rather than a validated clinical decision threshold. Baseline CAR also demonstrated good discriminative performance with an optimal cutoff value of 9.35, yielding the same sensitivity (73.7%) and a specificity of 83.1%.

The diagnostic performance of all evaluated biomarkers, including AUC values, optimal cutoff values, sensitivity, specificity, positive predictive value, and negative predictive value, is summarized in Table 4.

**Table 4:** Receiver operating characteristic analysis for prediction of osteomyelitis recurrence.

Marker	AUC	95% CI	Optimal cut-off	Sensitivity (%)	Specificity (%)	PPV (%)	NPV (%)
One-month CAR	0.862	0.750–0.946	1.41	73.7	87.3	60.9	92.5
Baseline CAR	0.857	0.766–0.932	9.35	73.7	83.1	53.8	92.2
One-month CRP	0.831	0.709–0.929	5.36	68.4	85.9	56.5	91.0
Baseline CRP	0.824	0.730–0.916	31.60	73.7	80.3	50.0	91.9
CCI	0.765	0.654–0.876	2.00	78.9	66.2	38.5	92.2
NLR at diagnosis	0.698	0.549–0.829	4.18	84.2	56.3	34.0	93.0

Cut-off values were determined using the Youden index.

PPV, positive predictive value; NPV, negative predictive value.

### Logistic regression analysis

One-month CAR was modeled as a continuous variable. The Box–Tidwell analysis showed no significant evidence of departure from linearity in the logit for one-month CAR (*p* = 0.223).

Variables significantly associated with recurrence in the univariable analyses were evaluated for inclusion in the multivariable model. Because only 19 recurrence events occurred, the final model was intentionally restricted to one-month CAR and Charlson Comorbidity Index to minimize the risk of overfitting.

Both one-month CAR and CCI remained independently associated with osteomyelitis recurrence within the parsimonious multivariable model. Within the present model, each one-unit increase in one-month CAR was associated with a 5.42-fold increase in the odds of recurrence (OR=5.42, 95% CI 2.38–12.36; *p* < 0.001), whereas each one-point increase in Charlson Comorbidity Index nearly doubled the odds of recurrence (OR=1.94, 95% CI: 1.27–2.96; *p* = 0.002). In an exploratory interaction analysis, the CAR   ×   CCI interaction term did not reach statistical significance (*p* = 0.078), providing no statistically significant evidence that the association between one-month CAR and recurrence differed according to comorbidity burden.

To assess whether the inclusion of vertebral osteomyelitis influenced the primary findings, a sensitivity analysis was performed after excluding the five patients with vertebral involvement. The association between one-month CAR and recurrence remained essentially unchanged (OR=5.33, 95% CI 2.33–12.20; *p* < 0.001), as did the association with CCI (OR=1.95, 95% CI 1.27–3.01; *p* = 0.002). These findings indicate that inclusion of the vertebral cases did not materially influence the primary multivariable results.

**Figure 6:**
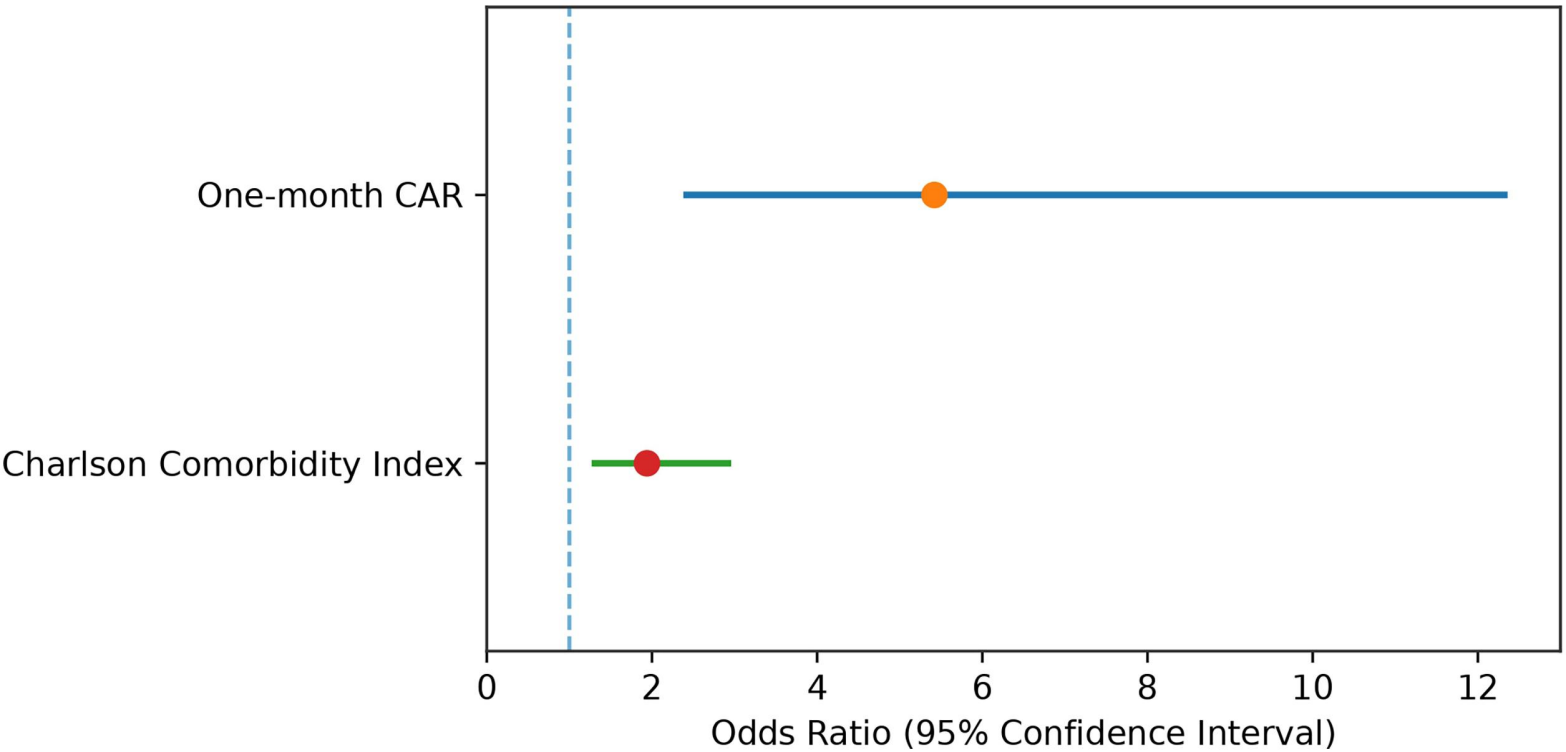
Forest plot showing adjusted associations of one-month CAR and Charlson Comorbidity Index with osteomyelitis recurrence in the parsimonious multivariable logistic regression model.

The results of the univariable and multivariable logistic regression analyses are presented in Table 5, and the adjusted effect estimates are illustrated in Figure 6.

**Table 5:** Multivariable logistic regression analysis of factors associated with osteomyelitis recurrence.

Variable	OR	95% CI	*p*-value
One-month CAR	5.42	2.38–12.36	**<0**.**001**
Charlson Comorbidity Index	1.94	1.27–2.96	**0**.**002**

A parsimonious model including one-month CAR and Charlson Comorbidity Index was used because of the limited number of recurrence events.

Bold values indicate statistical significance (*p* < 0.05).

## Discussion

The present study demonstrated that the C-reactive protein-to-albumin ratio (CAR) measured approximately 1 month after surgical treatment was independently associated with osteomyelitis recurrence after adjustment for CCI within the present parsimonious model. Rather than simply reflecting the severity of infection at presentation, our findings suggest that postoperative CAR captures the biological response to treatment and therefore provides clinically relevant prognostic information during the early recovery period. In addition, the Charlson Comorbidity Index (CCI) also remained independently associated with recurrence within the present model, indicating that both the postoperative inflammatory response and baseline patient-related factors contribute to the risk of treatment failure. Taken together, these findings indicate that postoperative CAR may complement conventional clinical assessment by identifying patients at increased risk of recurrence during routine follow-up.

Despite substantial improvements in surgical debridement techniques, antimicrobial therapy, and multidisciplinary care, recurrence remains one of the most challenging aspects of osteomyelitis management. Reported recurrence rates vary considerably according to infection chronicity, anatomical location, host characteristics, and treatment strategy, but recurrence continues to represent a major cause of repeated surgery, prolonged antibiotic exposure, functional impairment, and healthcare costs (1–6). Consequently, identifying patients at increased risk during the early postoperative period remains clinically important. Although CRP and ESR are routinely used during follow-up, both markers have important limitations because they may remain elevated following surgery or normalize despite persistent low-grade infection (7–9). Therefore, attention has increasingly shifted toward composite inflammatory biomarkers that integrate different aspects of the host response rather than relying on isolated laboratory parameters.

Among these biomarkers, CAR has attracted considerable interest because it combines two biologically complementary variables. CRP is rapidly synthesized in response to pro-inflammatory cytokines and reflects ongoing systemic inflammation, whereas albumin decreases in response to inflammatory stress while also reflecting nutritional and metabolic status (14–16). Consequently, CAR incorporates both inflammatory burden and physiological reserve into a single parameter. Hypoalbuminemia in this setting should not be interpreted solely as a marker of nutritional status. During persistent inflammation, reduced albumin levels may also reflect suppressed hepatic albumin synthesis, increased capillary permeability, and systemic catabolic stress, thereby providing an indirect measure of impaired physiological reserve. By combining CRP, which reflects the magnitude of the inflammatory response, with albumin, which partly reflects the host's systemic reserve, CAR may therefore capture complementary biological dimensions relevant to failure of infection control. Our findings suggest that the same principle may also apply to osteomyelitis recurrence following surgical treatment.

An important observation of the present study was that postoperative CAR demonstrated a numerically slightly higher AUC than baseline CAR. Although both measurements were significantly associated with recurrence, laboratory values obtained approximately one month after surgery more likely reflect the combined effects of adequate debridement, antimicrobial therapy, and host recovery than the severity of the initial infection itself. Successful treatment is generally accompanied by rapid resolution of systemic inflammation together with gradual normalization of albumin concentrations. In contrast, persistent elevation of CAR during early follow-up may indicate ongoing inflammatory activity resulting from residual bacterial biofilm, insufficient source control, impaired vascularity, or an inadequate host response before obvious clinical recurrence develops (23, 25, 26). Biofilm-associated organisms may persist on devitalized bone or implant surfaces despite surgical debridement and systemic antimicrobial therapy. Such persistent local infection may sustain a low-grade systemic inflammatory response, potentially contributing to persistently elevated CRP and CAR during early postoperative follow-up even in the absence of overt clinical deterioration. Achieving adequate local infection control remains challenging in osteomyelitis, particularly in the presence of devitalized bone, impaired vascularity, and biofilm. Consequently, various local antimicrobial delivery strategies, including antibiotic-loaded spacers, have been investigated to achieve high antimicrobial concentrations at the infection site. Experimental studies comparing locally delivered antimicrobial agents, including rifaximin- and teicoplanin-based approaches, further illustrate the ongoing efforts to optimize local infection control in osteomyelitis 27. This observation is also reflected by the high negative predictive value of postoperative CAR in the present cohort, although predictive values should be interpreted in the context of disease prevalence.

Although an AUC of 0.862 does not indicate perfect discrimination, it represents good predictive performance for a biomarker derived from routinely available laboratory tests. Although one-month CAR yielded a numerically higher AUC than one-month CRP (0.862 vs. 0.831), this difference was not statistically significant in the DeLong comparison (*p* = 0.223). Therefore, the present findings do not establish superiority of CAR over CRP in terms of discriminatory performance. Rather, CAR may provide complementary information by integrating inflammatory activity with albumin-related physiological status into a single routinely available parameter. Given the multifactorial nature of osteomyelitis recurrence, postoperative CAR should be viewed as a tool for risk stratification rather than a standalone predictor of treatment failure. Accordingly, its greatest clinical value is likely to be achieved when interpreted alongside clinical assessment, microbiological findings, and imaging studies.

Interest in CAR within orthopedic surgery has increased rapidly over recent years. Previous studies have reported promising diagnostic performance in prosthetic joint infection, diabetic foot infection, fracture-related infection, and other musculoskeletal disorders (20–22). However, most available reports have focused primarily on differentiating infected from non-infected patients at presentation. Evidence regarding the prognostic significance of postoperative CAR remains scarce. Unlike previous studies evaluating diagnostic accuracy, our investigation specifically examined the ability of an early postoperative measurement to predict subsequent recurrence after surgical treatment. This distinction is clinically relevant because recurrence risk assessment represents a different clinical problem from establishing the initial diagnosis. Rather than identifying active infection, postoperative CAR may help recognize patients whose inflammatory recovery deviates from the expected postoperative course despite apparently successful treatment.

Interestingly, leukocyte-derived inflammatory indices demonstrated considerably weaker prognostic performance than CAR during postoperative follow-up. Although baseline NLR and MLR were significantly higher in patients who subsequently developed recurrence, these differences largely disappeared at one month, whereas CAR remained strongly associated with recurrence. Leukocyte counts are influenced by numerous perioperative factors, including surgical stress, transient physiological responses, hydration status, and short-term fluctuations in immune cell populations (10–13). In contrast, CAR integrates both acute inflammatory activity and the patient's systemic metabolic response, potentially making it a more stable indicator of ongoing pathological inflammation. This observation may explain why one-month CAR remained associated with recurrence after adjustment for baseline comorbidity burden within the present model.

Besides postoperative inflammatory status, host-related factors also played an important role in recurrence risk. CCI also remained independently associated with recurrence within the multivariable model, emphasizing that successful infection eradication depends not only on adequate surgical treatment but also on the patient's ability to recover from infection. Patients with multiple comorbidities frequently exhibit impaired immunity, compromised tissue perfusion, delayed wound healing, and reduced physiological reserve, all of which may facilitate bacterial persistence or reinfection (1, 2, 23, 25). The observed associations of both CAR and CCI therefore suggest that postoperative inflammatory recovery and baseline host condition should be considered complementary rather than competing determinants of clinical outcome. Exploratory interaction analysis did not demonstrate a statistically significant CAR   ×   CCI interaction (*p* = 0.078). Thus, the present data do not provide sufficient evidence that the association between postoperative CAR and recurrence is modified by baseline comorbidity burden. However, given the limited number of recurrence events, this analysis may have been underpowered to detect effect modification, and the possibility of an interaction should be reassessed in larger cohorts.

From a clinical perspective, the findings of the present study have several practical implications. The laboratory parameters required to calculate CAR are inexpensive, universally available, and routinely obtained during postoperative follow-up without additional cost or patient burden. Therefore, incorporating CAR into routine surveillance protocols would be straightforward in most orthopedic centers. However, the one-month CAR cutoff of 1.41 identified in the present study should be interpreted as an exploratory, cohort-derived threshold rather than a validated clinical decision threshold. Although bootstrap internal validation demonstrated relatively stable discriminatory performance, the wider bootstrap distribution of the optimal cutoff indicates uncertainty regarding the precise threshold. Therefore, external validation in independent cohorts is required before the value of 1.41 can be adopted for clinical decision-making. Pending such validation, a one-month CAR value above 1.41 should not by itself prompt prolongation of antimicrobial therapy or repeat surgery. Rather, it may be considered a warning signal warranting closer clinical reassessment, including evaluation of wound status and symptoms, repeat inflammatory markers, and, when clinically indicated, earlier imaging and microbiological investigation. Persistent clinical, laboratory, or imaging evidence suggestive of ongoing infection should then prompt multidisciplinary reassessment of antimicrobial treatment and consideration of repeat surgical evaluation. Importantly, CAR should not be regarded as a standalone diagnostic test or a substitute for clinical evaluation, microbiological assessment, or imaging studies. Instead, it should be interpreted as an adjunctive marker that may help identify patients requiring closer follow-up, earlier imaging, or multidisciplinary reassessment of antimicrobial and surgical management. Conversely, the high negative predictive value observed in our cohort suggests that a low postoperative CAR may identify patients at lower risk of recurrence; however, this potential application also requires validation in independent cohorts before CAR-based follow-up strategies can be recommended.

An important strength of the present study is that it reflects the heterogeneous nature of osteomyelitis encountered in routine orthopedic practice. Rather than restricting the analysis to a single subtype, we included patients with diabetic foot, implant-related, post-traumatic, vascular insufficiency-associated, and postoperative osteomyelitis involving different anatomical locations. Although biological heterogeneity may reduce statistical precision, it enhances the external validity of the findings by reflecting the spectrum of osteomyelitis encountered in daily orthopedic practice. Despite these differences in etiology and localization, neither infection type nor anatomical site differed significantly between patients with and without recurrence, whereas postoperative CAR remained strongly associated with recurrence. Consistent with this observation, exploratory analyses showed no significant differences in postoperative CAR according to implant-associated etiology or anatomical location, suggesting that the observed association between CAR and recurrence was not obviously attributable to these factors. Moreover, sensitivity analysis excluding patients with vertebral osteomyelitis yielded nearly identical effect estimates for postoperative CAR, suggesting that the primary association was not driven by the inclusion of vertebral cases. This observation suggests that CAR may capture a common systemic inflammatory response associated with persistent infection regardless of the underlying cause. This hypothesis, however, requires confirmation in larger disease-specific cohorts. Considering that orthopedic surgeons frequently manage diverse forms of osteomyelitis rather than highly selected patient populations, our findings may therefore be more representative of real-world clinical practice than studies restricted to a single disease entity. Nevertheless, subgroup-specific conclusions cannot be drawn from the present data because the study was not powered for analyses according to osteomyelitis etiology or anatomical location. Future prospective studies involving more homogeneous patient populations are warranted to determine whether the prognostic value of postoperative CAR differs across specific osteomyelitis subtypes.

Our findings are broadly consistent with previous reports identifying host-related factors as major determinants of osteomyelitis recurrence. Garcia Del Pozo et al. identified host-related factors and comorbidity burden as major determinants of relapse following treatment of long-bone osteomyelitis, emphasizing that successful infection control depends on both adequate surgical management and patient characteristics (23). More recently, advances in the understanding of musculoskeletal infections have highlighted the complex interaction between bacterial biofilm formation, impaired vascularity, host immune dysfunction, and local tissue damage as key mechanisms contributing to recurrence (25, 26). Our findings are consistent with these concepts. While CCI likely reflects the patient's intrinsic susceptibility to recurrent infection, postoperative CAR appears to reflect the biological response to treatment. The observed associations of both variables indicate that recurrence risk is multifactorial and cannot be adequately explained by either host factors or inflammatory biomarkers alone.

Several limitations should be acknowledged when interpreting the results of this study. The retrospective single-center design introduces the possibility of selection bias and precludes causal inference. The relatively small number of recurrence events limited the complexity of the multivariable model and precluded reliable subgroup analyses, particularly according to microbiological species and osteomyelitis etiology. Although a parsimonious model was intentionally adopted to reduce the risk of overfitting, external validation in larger prospective cohorts is warranted. Detailed treatment-related variables, including the duration and type of antimicrobial therapy, specific surgical strategy, implant retention or removal, and use of local antimicrobial treatment, were not incorporated into the present analysis. Furthermore, vascular status was not systematically recorded as a separate variable across the entire cohort. Therefore, residual confounding related to differences in surgical and antimicrobial management and underlying vascular status cannot be excluded. The inclusion of different osteomyelitis etiologies and anatomical locations resulted in heterogeneous treatment strategies. While this approach improves the generalizability of the findings by reflecting routine clinical practice, it limits disease-specific conclusions. Inflammatory biomarkers were assessed at a single postoperative time point approximately one month after surgery. Serial measurements at predefined postoperative intervals may better characterize CAR kinetics, determine whether the trajectory of CAR provides additional prognostic information beyond a single measurement, and identify the optimal postoperative time point for recurrence risk stratification. Finally, the optimal one-month CAR cutoff of 1.41 was derived from the same relatively small cohort in which its diagnostic performance was evaluated. Although bootstrap internal validation supported the stability of the overall discriminatory performance, variability in the bootstrap-derived optimal cutoff indicates that the precise threshold remains uncertain. Therefore, the proposed cutoff should not be considered a validated clinical threshold, and external validation in larger independent cohorts is required. The present findings should consequently be regarded as hypothesis-generating and require external validation in larger, independent, multicenter cohorts.

Despite these limitations, the study has several important strengths. To our knowledge, this is among the few studies specifically evaluating the prognostic value of early postoperative CAR for predicting osteomyelitis recurrence after surgical treatment rather than its diagnostic performance at presentation. In addition, all patients underwent standardized surgical management at a single institution, recurrence was defined using objective clinical, microbiological, radiological, and surgical criteria, and multiple inflammatory biomarkers were evaluated simultaneously, allowing direct comparison of their predictive performance. The incorporation of receiver operating characteristic analysis together with multivariable logistic regression provides complementary assessment of the discriminatory performance and adjusted association of postoperative CAR with recurrence. Furthermore, the use of routinely available laboratory parameters enhances the potential applicability of our findings across different healthcare settings without increasing costs or requiring specialized testing.

## Conclusion

The present study demonstrated that the C-reactive protein-to-albumin ratio measured approximately one month after surgical treatment was independently associated with osteomyelitis recurrence after adjustment for comorbidity burden within the present model. These findings suggest that postoperative CAR may provide clinically meaningful information for early risk stratification, although its discriminative performance was not statistically superior to that of one-month CRP. Given its simplicity, low cost, and widespread availability, CAR appears to be a promising adjunctive biomarker for postoperative surveillance of patients with osteomyelitis. Nevertheless, larger prospective multicenter studies are required to validate the proposed cutoff values and determine whether CAR-guided follow-up strategies can improve long-term clinical outcomes.

## Data Availability

The raw data supporting the conclusions of this article will be made available by the authors, without undue reservation.
